# Association between prediagnostic leukocyte telomere length and breast cancer risk: the Singapore Chinese Health Study

**DOI:** 10.1186/s13058-019-1133-0

**Published:** 2019-04-17

**Authors:** Hamed Samavat, Xiaoshuang Xun, Aizhen Jin, Renwei Wang, Woon-Puay Koh, Jian-Min Yuan

**Affiliations:** 1 Division of Cancer Control and Population Sciences, UPMC Hillman Cancer Center, University of Pittsburgh, UPMC Cancer Pavilion, Suite 4C, 5150 Centre Avenue, Pittsburgh, PA 15232 USA; 2 Department of Epidemiology, Graduate School of Public Health, University of Pittsburgh, Pittsburgh, PA USA; 3 Heath Services and Systems Research, Duke-NUS Medical School, Singapore, Singapore; 4 Saw Swee Hock School of Public Health, National University of Singapore, Singapore, Singapore

**Keywords:** Telomere length, Blood leukocytes, Breast cancer, Prospective cohort study, Risk factors, Biomarkers

## Abstract

**Background:**

Telomeres and telomerase play key roles in the chromosomal maintenance and stability. Recent epidemiological studies have shown that longer telomeres are associated with increased risk of several cancer types. However, epidemiological data for telomere length and risk of breast cancer are sparse.

**Methods:**

We prospectively studied the association between telomere length and risk of breast cancer in 14,305 middle-aged or older Chinese women of the Singapore Chinese Health Study including 442 incident breast cancer cases after 12.3 years of follow-up. Relative telomere length in peripheral blood leukocytes was quantified using a validated monochrome multiple quantitative polymerase chain reaction method. The Cox proportional hazard regression method was used to estimate hazard ratios (HRs) and the corresponding 95% confidence intervals (CIs) for breast cancer associated with longer telomeres after adjustment for potential confounders.

**Results:**

Longer telomeres were significantly associated with higher risk of breast cancer in a dose-dependent manner (*P*_trend_ = 0.006); the highest quartile of telomere length was associated with a statistically significant 47% higher risk of breast cancer compared with the lowest quartile of telomere length after the adjustment for age and other known risk factors for breast cancer (HR_Q4 vs Q1_ = 1.47, 95% CI = 1.11, 1.94).

**Conclusions:**

The findings of the present study support the hypothesis that longer telomeres may be a risk factor for breast cancer. Telomere length in peripheral blood leukocytes may be developed as a biomarker for breast cancer risk prediction.

**Electronic supplementary material:**

The online version of this article (10.1186/s13058-019-1133-0) contains supplementary material, which is available to authorized users.

## Introduction

Breast cancer is the most common cancer for women. Worldwide, 2.4 million new cases of breast cancer and 534,000 deaths from breast cancer occurred in 2015 [1]. It is estimated that breast cancer accounts for 1 in 3 new cancer cases in American women, and over 40,000 American women die from breast cancer in 2018 [2]. Previous epidemiological studies have established many risk factors for breast cancer including first-degree family history of breast cancer, early age at menarche, nulliparity, late age at first birth, late age at menopause, overweight or obesity, breast density, exogenous hormone use, and history of benign breast biopsy [3]. All these factors together explain up to 70% of breast cancer burden among postmenopausal women in the USA [4–6]. New predictors for breast cancer risk may help identify women at higher risk of breast cancer.

Telomeres are tandemly repeated sequences of TTAGGG located at the distal ends of linear chromosomes [7]. They play an essential role in maintaining the structural integrity of chromosomes and regulating cell replication through preventing DNA double-strand breaks, end-to-end chromosome fusions, and degradation [8]. Owing to the incomplete DNA replication at the end of chromosome, telomeres shorten by 50–200 bp during each cell division [9]. Progressive telomere shortening often leads to genomic instability and eventually results in apoptosis or cellular senescence [10]. This progress is considered as a tumor suppressor mechanism, as it limits the number of cell replication cycles [7]. However, if cells bypass senescence due to dysfunctional checkpoint pathways, the telomeres will continue to shorten, driving chromosome fusion and genomic instability. Survivors of this telomere crisis then maintain telomeres, a cancer hallmark, by upregulating telomerase in most cases [11]. Thus, these surviving cells with longer telomeres have a replicative advantage [12], consequently undergo more cell divisions prior to telomere crisis, resulting in the increased likelihood for acquiring mutations that drive malignant transformation [12].

In the last decade, a growing number of studies have examined the association between telomere length and the risk of cancer. To date, 8 retrospective case-control studies [13–20] and 5 prospective studies [20–24] have been published on the relationship between telomere length and risk of breast cancer where their results have been mixed. Some studies [13, 14] reported a positive while others [15, 19–21] found a negative association between telomere length and breast cancer. The remaining studies showed a null association [16–18, 20, 22–24]. In addition, recent prospective epidemiological studies have shown that longer telomeres are associated with increased risk of several cancer types including lung cancer [25, 26], prostate cancer [27], and pancreatic cancer [28]. However, the data from prospective epidemiological studies on telomere length and the risk of breast cancer are sparse. Utilizing the Singapore Chinese Health Study, a prospective study of more than 60,000 middle-aged or older Chinese men and women in Singapore, we investigated the association between telomere length and the risk of developing breast cancer.

## Materials and methods

### Study population

The present study was based on the data from the Singapore Chinese Health Study, a population-based prospective cohort study with original aims of investigating the role of diet, environmental exposures, and genetic factors on the etiology of cancer and other chronic diseases among Chinese in Singapore. The Institutional Review Boards of the National University of Singapore and the University of Pittsburgh approved the study. Detailed information on study design and methods has been described elsewhere [29]. Briefly, a trained interviewer administered an in-person interview to each consented participant using a structured questionnaire that solicited information on demographics, body weight and height, lifetime use of tobacco, current physical activity, menstrual and reproductive history (for women only), medical history, and family history of cancer. All baseline interviews to 63,257 participants were completed during 1993–1998.

The first follow-up interview was conducted in 1999–2003 to all surviving cohort participants. A total of 52,326 (90.6%) surviving participants completed the follow-up interview that updated information on cigarette smoking, alcohol drinking, history of respiratory diseases and other medical conditions, medication use, current body weight and height, and current menopausal status (for women).

Blood and urine samples were initially collected from a 3% random sample of cohort participants which began in April 1994. We expanded the biospecimen collection to all surviving cohort participants who consented for blood and urine donation at the end of the first follow-up interview. By the end of 2005, 28,346 (57%) of all eligible participants provided blood specimens. Blood components (plasma, serum, buffy coat, and red blood cells) were then stored at − 80 ^°^C for future analyses.

The present study included 14,305 women who completed the baseline and first follow-up interviews and donated a blood sample. These women were younger and more educated, otherwise comparable with women who did not provide blood samples (*n* = 20,998) in terms of body mass index, alcohol consumption, cigarette smoking, age when period became regular, family history of breast cancer, and number of hours slept per day (see Additional file 1: Table S1).

### Assessment of breast cancer cases

All incident cancer cases and death among cohort participants were identified by annual record linkage analysis with the databases of the Singapore Cancer Registry and the Birth and Death Registry, respectively [30]. According to the latest record, only 56 (< 0.1%) of the total 63,257 original cohort subjects were unknown to their cancer or vital status due to migration out of Singapore. Breast cancer was defined as the tenth revision of the International Classification of Diseases (ICD) and Related Health Problems codes C50.0–C50.9. As of December 31, 2015, after a mean follow-up period of 12.3 years, 442 women who were free of cancer at the time of blood collection developed breast cancer, which were included in the present analysis.

### Laboratory methods (for telomere length measurement)

Genomic DNA was extracted from the buffy coat in the peripheral blood sample using QIAamp 96 DNA Blood Kits (Qiagen, Valencia, CA) according to the manufacturer’s protocol. Relative telomere length was measured by comparing the ratio of telomere repeat number (*T*) to a single copy gene number for albumin (*S*) in the experimental sample to a standardized reference sample values using multiplexed quantitative polymerase chain reaction (PCR) method developed by Cawthon [31]. This simple and rapid method allowed us to use smaller amounts of DNA to get a relative telomere length and achieve a large population [32]. The experimental sample and reference sample kept the same relative quantity of a single copy gene and telomere repeats by controlling the number of PCR cycles needed to generate a given number of PCR product. PCR reaction was set up in the 96-well plate in the Bio-Rad MyiQ Single-Color Real-Time PCR Detection System by aliquoting 15 μL master mix and 10 μL of experimental DNA sample into each reaction well. The reference standard DNA curve was made from a pooled sample of 77 participants from the Singapore Chinese Health Study identified in a previous study. The reference samples were serially diluted in 5 concentrations in every 96-well plate to provide a relative quantitation. All experimental samples were assayed in duplicate, and the average value of the 2 replicates was used for the final analysis for each subject. The mean coefficient of variation, as a measure of reproducibility, of all technical sample duplicates for telomere length in the present study was 3.5%.

### Statistical analysis

Before the statistical analysis, we reconstructed the variables for BMI, cigarette smoking, alcohol consumption, menopausal status and age at menopause, use of oral contraceptives, and use of hormonal therapy based on both responses by study participants at baseline and follow-up 1 interviews. *t* test and *χ*^2^ test were performed to compare the distributions of the selected variables in continuous and discrete values, respectively, between breast cancer cases and non-cancer cases. Analysis of covariance (ANCOVA) was also used to examine the difference in mean relative telomere lengths by the levels of BMI, smoking status, alcohol consumption, and menstrual and reproductive history in all women after adjusted for age at sample collection and father dialect.

The Cox proportional hazard regression method was used to examine the association between relative telomere length and the risk of breast cancer. Person-years for each study participant at risk were calculated from the date of blood sample collection to the date of breast cancer diagnosis, death, migration out of Singapore, or 31 December 2015, whichever occurred first. Study subjects were grouped into quartile levels of telomere length according to the following interquartile range of relative telomere lengths: 0.73–0.85 (Q1), 0.92–0.99 (Q2), 1.06–1.13 (Q3), and 1.23–1.40 (Q4). The magnitude of the association between telomere length and breast cancer risk was measured by hazard ratios (HRs) and corresponding 95% confidence intervals (95% CIs). Tests for linear trend were carried out by taking quartiles of telomere length as an ordinal variable in the Cox model. The proportional hazard assumption was examined using the Schoenfeld method [33] that did not show any violation to the proportionality assumption.

Two sets of Cox regression models were employed in assessing the association between telomere length and breast cancer risk. The first model included age (years) at blood draw and dialect group (Hokkien or Cantonese) as covariates whereas the second model included additional covariates as follows: level of education (no formal education, primary school, or secondary school and above), BMI (< 23.0 kg/m^2^ or ≥ 23.0 kg/m^2^ according to the recommendation by the World Health Organization (WHO) for Asian populations [34]), age at first live birth (< 20, 21–25, 26–30, or ≥ 31 years), number of live births (0, 1–2, 3–4, or ≥ 5), age at menopause (≤ 49, 50–54, or ≥ 55 years), use of hormone therapy (never, ever, or current), use of oral contraceptives (no or yes), family history of breast cancer (no or yes), smoking status (never, former, or current smoker), alcohol consumption (non-drinker, < 7, or ≥ 7 drinks per week), weekly vigorous work or strenuous sports (no or yes), and number of hours of sleep.

Effect modification on the association between telomere length and breast cancer risk was examined for age and BMI based on model 2, which included a product term of the modifier and telomere length. Sensitivity analysis was conducted using the same multivariate Cox regression model (model 2) to examine if the association between telomere length and risk of breast cancer was similar for women with shorter (e.g., < 5 years) and those with longer (e.g., ≥ 5 years) follow-up after blood collection.

All statistical analyses were conducted using SAS 9.4 software package (SAS Institute, Cary, NC). All *P* values reported are two sided. *P* < 0.05 was considered statistically significant.

## Results

The present analysis included 442 incident breast cancer cases and 13,863 women who were free of breast cancer (non-cancer cases) at the end of follow-up for the present analysis. Fifty-three percent of women were Hokkiens and 47% were Cantonese. The mean age at breast cancer diagnosis was 61.1 [standard deviation (SD) 7.4]. The median time interval from the date of blood collection to the date of breast cancer diagnosis was 6.3 years (range from < 1 month to 18.4 years).

Table 1 shows the distributions of participants’ characteristics and their association with risk of breast cancer. High levels of BMI and education, early age when menstrual period became regular, late age at first live birth, nulliparous or fewer number of live births, and late age at menopause were statistically significantly or borderline significantly associated with higher risk of breast cancer. Alcohol consumption, cigarette smoking status, weekly vigorous work or strenuous sports, use of oral contraceptives, use of hormonal therapy, number of hours of sleep, or familial history of breast cancer were not significantly associated with risk of breast cancer.

**Table 1:** Distributions of participants’ characteristics and their corresponding hazard ratio for breast cancer among women, the Singapore Chinese Health Study

Characteristics	Non-cancer cases, *N* (%)	Breast cancer cases, *N* (%)	HR^1^ (95% CI)	*P* ^1^
Body mass index^§^, kg/m^2^				
< 18.5	992 (7.2)	24 (5.4)	1.00 (ref)	0.002
18.5 to < 23.0	5700 (41.1)	159 (36.0)	1.09 (0.71, 1.68)	
23.0 to < 27.5	5521 (39.8)	195 (44.1)	1.43 (0.93, 2.18)	
≥ 27.5	1650 (11.9)	64 (14.5)	1.56 (0.97, 2.49)	
Level of education				
No formal education	4471 (32.3)	109 (24.7)	1.00 (ref)	0.013
Primary school	5840 (42.1)	194 (43.9)	1.24 (0.97, 1.58)	
Secondary school and above	3552 (25.6)	139 (31.5)	1.42 (1.08, 1.86)	
Alcohol consumption^§^ (drinks/week)				
None	12,403 (89.5)	385 (87.1)	1.00 (ref)	0.15
< 7	1283 (9.3)	48 (10.9)	1.14 (0.84, 1.53)	
≥ 7	177 (1.3)	9 (2.0)	1.57 (0.81, 3.05)	
Physical activity (weekly)				
No	10,103 (72.9)	318 (72.0)	1.00 (ref)	0.91
Yes	3760 (27.1)	124 (28.1)	1.01 (0.82, 1.25)	
Smoking status^§^				
Never	12,639 (91.2)	414 (93.7)	1.00 (ref)	0.39
Former/current	1224 (8.8)	28 (6.3)	0.84 (0.57, 1.24)	
Age when period became regular^§^				
< 13	1918 (13.8)	70 (15.8)	1.61 (1.09, 2.38)	0.016
13–14	4977 (35.9)	175 (39.6)	1.61 (1.14, 2.26)	
15–16	4435 (31.9)	143 (32.4)	1.53 (1.08, 2.16)	
≥ 17	2045 (14.8)	42 (9.5)	1.00 (ref)	
Never regular	488 (3.5)	12 (2.7)	1.13 (0.59, 2.15)	
Age at first live birth				
< 20.0	2363 (17.1)	57 (12.9)	1.00 (ref)	0.005
21.0–25.0	5195 (37.5)	162 (36.7)	1.19 (0.88, 1.61)	
26.0–30.0	3840 (27.7)	122 (27.6)	1.15 (0.84, 1.59)	
≥ 31	1475 (10.6)	51 (11.5)	1.27 (0.86, 1.86)	
Nulliparous	990 (7.1)	50 (11.3)	1.90 (1.29, 2.80)	
Number of live births				
0	977 (7.1)	50 (11.3)	1.00 (ref)	0.0002
1–2	4213 (30.4)	159 (36.0)	0.70 (0.51, 0.97)	
3–4	5591 (40.3)	157 (35.5)	0.54 (0.39, 0.74)	
≥ 5	3082 (22.2)	76 (17.2)	0.54 (0.37, 0.79)	
Age at menopause^§^				
≤ 49	2371 (17.1)	54 (12.2)	1.00 (ref)	0.05
50–54	3284 (23.7)	92 (20.8)	1.21 (0.86, 1.69)	
≥ 55	8208 (59.2)	296 (67.0)	1.43 (1.00, 2.05)	
Family history of breast cancer				
No	13,631 (98.3)	434 (98.2)	1.00 (ref)	0.96
Yes	232 (1.7)	8 (1.8)	1.02 (0.51, 2.05)	
Use of hormone therapy^§^				
Never	11,543 (83.3)	347 (78.5)	1.00 (ref)	0.10
Ever	1540 (11.1)	62 (14.0)	1.26 (0.96, 1.65)	
Current	780 (5.6)	33 (7.5)	1.21 (0.84, 1.74)	
Use of oral contraceptive				
Never	9586 (69.2)	302 (68.3)	1.00 (ref)	0.70
Former/current	4277 (30.9)	140 (31.7)	0.98 (0.78, 1.18)	
Sleeping hours				
≤ 6 h	4737 (34.2)	134 (30.3)	0.84 (0.69, 1.04)	0.48
7–8 h	8218 (59.3)	285 (64.5)	1.00 (ref)	
≥ 8 h	908 (6.6)	23 (5.2)	0.75 (0.49, 1.15)	

Values are presented as frequency (%) or mean (SD). Percentages may not add up to 100% due to rounding

*CI* confidence intervals, *HR* hazard ratio

^§^Updated with data from the first follow-up interview

^1^Hazard ratios and *P* values for linear trend were derived from the Cox proportional hazard regression models that also included age at blood collection and dialect (Hokkien or Cantonese)

The mean relative telomere lengths by observed or potential risk factors for breast cancer are shown in Table 2. As reported previously [26], age was inversely correlated with telomere length (*r* = − 0.24; *P* ≤ 0.0001) and accounted for 5.5% of the telomere length variation (*P* < 0.0001). After adjustment for age and father dialect as the cohort recruitment criteria factors, a greater number of live births, postmenopausal status, Hokkien dialect, and shorter duration of sleep were significantly or borderline significantly associated with longer telomeres.

**Table 2:** Mean relative telomere length by characteristics of women, the Singapore Chinese Health Study

Variables	Age at blood draw	Number	Telomere length (95% CI)	*P* ^1^
Age at sample collection, years				
45 to < 55	52.5	2452	1.11 (1.10, 1.12)	< 0.0001
55 to < 60	56.8	3528	1.09 (1.08, 1.09)	
60 to < 65	62.0	3173	1.04 (1.03, 1.05)	
≥ 65	70.9	5152	0.98 (0.97, 0.99)	
Body mass index, kg/m^2^				
< 18.5	63.6	1016	1.04 (1.02, 1.05)	0.21
18.5 to < 23.0	61.8	5859	1.05 (1.04, 1.05)	
23.0 to < 27.5	62.6	5716	1.05 (1.04, 1.05)	
≥ 27.5	62.3	1714	1.04 (1.03, 1.05)	
Level of education				
No formal education	66.4	4580	1.05 (1.04, 1.06)	0.16
Primary school	61.6	6034	1.04 (1.04, 1.05)	
Secondary school and above	58.3	3691	1.04 (1.03, 1.05)	
Dialect group				
Cantonese	62.5	7519	1.04 (1.04, 1.05)	0.08
Hokkien	62.0	6786	1.05 (1.04, 1.05)	
Alcohol consumption, drinks/week				
None	62.5	12,788	1.04 (1.04, 1.05)	0.41
< 7	60.6	1331	1.05 (1.04, 1.06)	
≥ 7	62.0	186	1.06 (1.03, 1.09)	
Physical activity (weekly)				
No	62.4	10,421	1.04 (1.04, 1.05)	0.71
Yes	61.9	3884	1.04 (1.04, 1.05)	
Smoking status				
Never	61.8	13,053	1.04 (1.04, 1.05)	0.87
Former	68.3	570	1.04 (1.02, 1.06)	
Current	66.7	682	1.04 (1.03, 1.06)	
Age when period became regular				
< 13	59.1	1988	1.04 (1.04, 1.05)	0.77
13–14	61.5	5152	1.04 (1.04, 1.05)	
15–16	63.5	4578	1.05 (1.04, 1.05)	
≥ 17	64.7	2087	1.05 (1.04, 1.05)	
Never regular	61.7	500	1.06 (1.04, 1.08)	
Age at first live birth				
< 20.0	65.6	2420	1.05 (1.04, 1.06)	0.42
21.0–25.0	62.5	5357	1.04 (1.04, 1.05)	
26.0–30.0	60.8	3962	1.04 (1.03, 1.05)	
≥ 31	61.0	1526	1.05 (1.04, 1.06)	
Nulliparous	61.3	1040	1.04 (1.03, 1.06)	
Number of live births				
0	61.2	1027	1.04 (1.03, 1.05)	0.07
1–2	59.3	4327	1.04 (1.03, 1.05)	
3–4	61.3	5748	1.04 (1.04, 1.05)	
≥ 5	68.6	3158	1.05 (1.05, 1.06)	
Menopausal status				
Premenopausal	52.6	955	1.03 (1.02, 1.05)	0.09
Postmenopausal	63.0	13,350	1.05 (1.04, 1.05)	
Age at menopause				
≤ 49	69.4	2425	1.05 (1.04, 1.06)	0.97
50–54	68.8	3376	1.04 (1.04, 1.05)	
≥ 55	57.7	8504	1.04 (1.04, 1.05)	
Family history of breast cancer				
No	62.3	14,065	1.04 (1.04, 1.05)	0.20
Yes	60.6	240	1.06 (1.03, 1.09)	
Use of hormone therapy				
Never	63.0	11,890	1.06 (1.05, 1.08)	0.82
Ever	59.6	1602	1.04 (1.03, 1.05)	
Current	57.4	813	1.04 (1.04, 1.05)	
Use of oral contraceptive				
Never	63.2	9888	1.05 (1.04, 1.05)	0.23
Former/current	60.2	4417	1.04 (1.03, 1.05)	
Sleeping hours				
≤ 6 h	63.2	4871	1.05 (1.04, 1.05)	0.04
7–8 h	61.8	8503	1.04 (1.04, 1.05)	
≥ 8 h	62.4	931	1.03 (1.01, 1.04)	

^1^Values are least-square means (95% confidence intervals), and *P* values were derived from ANCOVA with adjustment for age at blood collection and dialect group

Overall mean relative telomere length measure was slightly higher in breast cancer cases (mean 1.07, SD 0.22) than those without breast cancer (mean 1.04, SD 0.23) (*P* = 0.012). Longer telomeres were significantly associated with a higher risk of breast cancer in a dose-dependent manner after adjustment for age and other potential confounders (*P*_trend_ = 0.006) (Table 3). Compared with the lowest quartile, the highest quartile of telomere length was associated with a statistically significant 47% higher risk of breast cancer (HR = 1.47; 95% CI = 1.11, 1.94). When women were stratified by age and BMI, the associations between telomere length and breast cancer risk were slightly stronger in younger women (< 60 years of age) and in overweight or obese women (BMI ≥ 23 kg/m^2^) (Table 4). However, the differences in the magnitude of these associations between the contrasting groups were not statistically significant (*P*_trend_ > 0.30).

**Table 3:** Associations between relative telomere length and risk of breast cancer, the Singapore Chinese Health Study

Telomere length in quartile^§^	Cases	Person-years	HR^1^ (95% CI)	HR^2^ (95% CI)
Q1 (0.73–0.85)	84	43,035	1.00 (ref)	1.00 (ref)
Q2 (0.92–0.99)	106	44,093	1.21 (0.91, 1.62)	1.22 (0.91, 1.62)
Q3 (1.06–1.13)	120	44,312	1.35 (1.02, 1.79)	1.35 (1.01, 1.78)
Q4 (1.23–1.40)	132	45,174	1.45 (1.09, 1.91)	1.47 (1.11, 1.94)
*P*_trend_			0.008	0.006

*CI* confidence intervals, *HR* hazard ratio

^§^Numbers inside the parentheses are interquartile ranges

^1^Hazard ratios and *P* values were derived from the Cox proportional hazard regression model that included age at blood collection and dialect group (Hokkien or Cantonese)

^2^Hazard ratio and *P* values were derived from the Cox proportional hazard regression model that also included level of education (no formal education, primary school, or secondary school and above), BMI (< 18.5, 18.5 to < 23, 23 to < 27.5, 25.5+ kg/m^2^), age when period became regular, age at first live birth (< 10, 21–25, 26–30, or ≥ 31 years), number of live births (0, 1–2, 3–4, or ≥ 5), age at menopause (≤ 49, 50–54, or ≥ 55 years), use of hormone therapy (never, ever, or current), use of oral contraceptives (no or yes), family history of breast cancer (no or yes), smoking status (never, former, or current smoker), alcohol consumption (non-drinker, < 7 or ≥ 7 drinks per week), weekly vigorous work or strenuous sports (no or yes), and number of hours of sleep

**Table 4:** Associations between relative telomere length and risk of breast cancer stratified by age and body mass index, Singapore Chinese Health Study

Telomere length in quartile by stratification variable	Cases	Person-years	Adjusted HR^1^ (95% CI)	*P* interaction
Age (< 60 years)				
Q1 (shortest)	28	12,985	1.00 (reference)	0.30
Q2	41	17,826	1.05 (0.65, 1.70)	
Q3	64	20,853	1.38 (0.88, 2.15)	
Q4 (longest)	86	25,899	1.52 (0.99, 2.33)	
*P*_trend_			0.017	
Age (≥ 60 years)				
Q1 (shortest)	56	30,050	1.00 (reference)	
Q2	65	26,267	1.32 (0.92, 1.89)	
Q3	56	23,459	1.26 (0.87, 1.82)	
Q4 (longest)	46	19,275	1.27 (0.86, 1.88)	
*P*_trend_			0.25	
Body mass index (< 23.0 kg/m^2^)				
Q1 (shortest)	38	20,617	1.00 (reference)	0.45
Q2	44	21,321	1.12 (0.72, 1.73)	
Q3	46	21,377	1.14 (0.74, 1.76)	
Q4 (longest)	55	22,407	1.31 (0.86, 2.01)	
*P*_trend_			0.21	
Body mass index (≥ 23.0 kg/m^2^)				
Q1 (shortest)	46	22,417	1.00 (reference)	
Q2	62	22,772	1.32 (0.90, 1.94)	
Q3	74	22,935	1.51 (1.04, 2.19)	
Q4 (longest)	77	22,766	1.59 (1.09, 2.32)	
*P*_trend_			0.013	

*CI* confidence intervals, *HR* hazard ratio

^1^Hazard ratio derived from Cox proportional hazard regression model adjusted for age at sample collection, dialect group, level of education, BMI, age when period became regular, age at first live birth, number of live births, age at menopause, use of hormone therapy, use of oral contraceptives, family history of breast cancer, smoking status, alcohol consumption, weekly vigorous work or strenuous sports, and number of hours of sleep

We performed a sensitivity analysis by dividing breast cancer cases with various number of years from blood draw to cancer diagnosis to evaluate if the underlying subclinical disease progression had any impact on telomere length and so on the association between telomere length and risk of breast cancer (Table 5). For women with less than 5 years of follow-up, HR (95% CI) of breast cancer incidence for the highest compared to the lowest quartile of telomere length was 1.68 (1.05–2.69) (*P*_trend_ = 0.015). The corresponding figure for women with five or more years of follow-up was 1.35 (0.95–1.92) (*P*_trend_ = 0.12). However, *P* for heterogeneity in comparison of the two hazard ratios was not statistically significant (*P* = 0.47). As shown in Additional file 1: Table S2, when we limited the sensitivity analysis to breast cancer cases diagnosed during the first 2 years of follow-up, a similar result was found (*P* for heterogeneity = 0.32).

**Table 5:** Associations between relative telomere length and risk of breast cancer stratified by length of follow-up, Singapore Chinese Health Study

Telomere length in quartile by stratification variable	Cases	Person-years	Adjusted HR^1^ (95% CI)
Follow-up < 5 years			
Q1 (shortest)	29	17,272	1.00 (reference)
Q2	42	17,329	1.42 (0.89, 2.29)
Q3	58	17,298	1.95 (1.24, 3.06)
Q4 (longest)	50	17,353	1.68 (1.05, 2.69)
*P*_trend_			0.015
Follow-up ≥ 5 years			
Q1 (shortest)	55	25,763	1.00 (reference)
Q2	64	26,764	1.11 (0.77, 1.59)
Q3	62	27,014	1.04 (0.72, 1.50)
Q4 (longest)	82	27,821	1.35 (0.95, 1.92)
*P*_trend_			0.12

*CI* confidence intervals, *HR* hazard ratio

^1^Hazard ratio derived from Cox proportional hazard regression model adjusted for age at sample collection, dialect group, level of education, BMI, age when period became regular, age at first live birth, number of live births, age at menopause, use of hormone therapy, use of oral contraceptives, family history of breast cancer, smoking status, alcohol consumption, weekly vigorous work or strenuous sports, and number of hours of sleep

## Discussion

We investigated the association between white blood cell telomere length and breast cancer risk in a prospective study of 14,305 middle-aged or older Chinse women in Singapore. Our results showed that women in the top quarter of telomere length had a statistically significant 47% higher risk of breast cancer than women in the bottom quarter of telomere length after adjustment for age and other potential confounders. The positive association between telomere length and risk of breast cancer was in a dose-dependent manner.

We used peripheral blood leukocytes as the surrogate for breast tissue, which may have different telomere length. Studies have shown that telomere lengths varied considerably across different tissue types but were strongly correlated with one another within a person [35–37]. In addition, Daniali et al. [38] have reported that the rate of telomere shortening in somatic tissues was similar for different tissue types. There have been no studies that directly evaluated the correlation between telomere length in white blood cells and breast tissue.

Four prior case-cohort or nested case-control studies within prospective cohorts also reported mixed results. Three of the four studies in the USA and the UK found no significant association between telomere length and risk of breast cancer incidence [20, 22, 24]. The other study in a Chinese population reported that shorter telomeres were associated with significantly higher risk of breast cancer [21]. There has been only one prospective cohort study that examined telomere length and risk of breast cancer and other specific cancer sites [23]. The cohort included more than 65,000 Danish women and found that longer telomeres in peripheral leukocytes were associated with a higher but statistically non-significant risk of breast cancer [23]. Our study found a statistically significant association between long telomeres and high risk of breast cancer in Chinese women in Singapore. Previous studies suggested that the discrepancies in the associations between telomere length and disease risk might have resulted from different methods used for DNA extraction and/or telomere length measurement [39, 40]. In our study, we used the same Qiagen DNA extraction method and the same qPCR assay for telomere length as the 5 studies described above except for one study [20] that the DNA extraction method was not described. Thus, the discrepancy in the results of ours from previous studies may not be explained by the DNA extraction and telomere length measurement methods. The discrepancy may be due to the different study populations and/or study design. Our findings warrant further studies for confirmation.

To date, two Mendelian randomization studies [41, 42] have investigated the possible causal relation between genetic variants associated with telomere length and risk of different cancer types including breast cancer. These studies yielded a null association between telomere length-associated single nucleotide polymorphisms (SNPs) and risk of breast cancer. Although Mendelian randomization studies potentially minimized the potential environmental confounding factors on telomere length, they could have the drawbacks of pleiotropic effects and population stratification biases.

Little is known about the potential biological mechanisms through which telomere influences breast cancer risk. In general, cells with very short telomeres may induce replicative senescence or apoptosis, which suppress the proliferative potential of a cell and, thus, lend support to a tumor-suppressor activity [7, 43]. On the other hand, telomere lengthening in women with breast cancer or basal-type breast cancer cells has been associated with genetic variations in genes encoding telomere-related proteins [44, 45] and epigenetic silencing of miR-296 and miR-512 genes expression [46], respectively. The hormonal effect has also been suggested as another explanation for longer telomeres and increased risk of breast cancer since estrogen is directly implicated in activation of telomerase via effects on human telomerase reverse transcriptase (hTERT) promoter [47] and post-transcriptional modification through Akt-dependent phosphorylation of hTERT [48]. However, previous observational reports, either case-cohort [22] or case-control studies [13–15], found no significant association between telomere length and breast cancer risk by hormone receptor status yet large prospective cohort studies with quantified estrogen levels are required to shed light on the role of sex steroid hormones in the relationship with telomere length.

Several strengths of the current study are noteworthy. This is the first prospective cohort study conducted in an ethnically homogenous population in Eastern Asia. In addition, exposure assessment (i.e., telomere length measurement) was performed on samples from all cohort women who provided blood prior to the diagnosis of breast cancer; thus, reverse causality bias is not a concern in our findings. The study also took advantage of a long duration of follow-up. Finally, the Singapore population-based Cancer Registry, as a nationwide program, has been recording cancer cases for more than the last 50 years, and therefore, breast cancer case ascertainment should be essentially complete [49]. There are some limitations to the present study. Although we collected extensive information on known environmental and lifestyle risk factors for breast cancer prior to cancer diagnosis at study enrollment, we cannot rule out the possibility of residual confounding effects. Another limitation is the relatively small sample size of breast cancer cases, particularly in stratification analyses. We did not also evaluate telomere length in relation to estrogen concentrations or hormone receptor status in the present analysis since these data were only available for a very small subset of cases, and therefore, we had limited statistical power to detect a meaningful risk estimate. Lastly, these results may not be generalizable to other ethnic groups or premenopausal women since approximately 93% of the breast cancer cases in our study were postmenopausal. Our findings require confirmation in other large cohorts of the Asian population.

## Conclusions

In conclusion, the results of this study show that longer prediagnostic telomere length in blood leukocytes is significantly associated with an enhanced risk of breast cancer in a stepwise manner among a predominantly postmenopausal Asian women population. Our findings provide further evidence that leukocyte telomere length has the potential to act as a moderate risk factor for breast cancer. Whether telomere length can be implemented as a biomarker for breast cancer risk prediction warrants replication in additional large prospective cohort studies with diverse populations.

## Additional file

Additional file 1: **Tables S1.** Distributions of participants’ characteristics for women who donated blood samples for telomere length measurement and women who did not provide blood samples for telomere length measurement, the Singapore Chinese Health Study. **Table S2.** Associations between relative telomere length and risk of breast cancer stratified by length of follow-up, Singapore Chinese Health Study. (DOCX 21 kb)

## Abbreviations

ANCOVA: Analysis of covariance
BMI: Body mass index
CI: Confidence interval
HR: Hazard ratio
hTERT: Human telomerase reverse transcriptase
ICD: International Classification of Diseases
PCR: Polymerase chain reaction

## References

[1] Fitzmaurice C, Allen C, Barber RM, Barregard L, Bhutta ZA, Brenner H (2017). Global, regional, and national cancer incidence, mortality, years of life lost, years lived with disability, and disability-adjusted life-years for 32 cancer groups, 1990 to 2015: a systematic analysis for the Global Burden of Disease Study Global Burden. JAMA Oncol.

[2] Siegel RL, Miller KD, Jemal A (2018). Cancer statistics, 2018. CA Cancer J Clin.

[3] Mina LA, Storniolo AM, Kipfer HD, Hunter C, Ludwig KK (2016). Breast cancer prevention and treatment.

[4] Engmann NJ, Golmakani MK, Miglioretti DL, Sprague BL, Kerlikowske K, Breast Cancer Surveillance Consortium (2017). Population-attributable risk proportion of clinical risk factors for breast cancer. JAMA Oncol.

[5] Tamimi RM, Spiegelman D, Smith-Warner SA, Wang M, Pazaris M, Willett WC (2016). Population attributable risk of modifiable and nonmodifiable breast cancer risk factors in postmenopausal breast cancer. Am J Epidemiol.

[6] Seidman H, Stellman SD, Mushinski MH (1982). A different perspective on breast cancer risk factors: some implications of the nonattributable risk. CA Cancer J Clin.

[7] Blasco MA (2005). Telomeres and human disease: ageing, cancer and beyond. Nat Rev Genet.

[8] Aubert G, Lansdorp PM (2008). Telomeres and aging. Physiol Rev.

[9] Muraki K, Nyhan K, Han L, Murnane JP (2012). Mechanisms of telomere loss and their consequences for chromosome instability. Front Oncol.

[10] Campisi J. Cellular senescence as a tumor-suppressor mechanism. Trends Cell Biol. 2001;11:S27-31. 10.1016/s0962-8924(01)02151-1 11684439

[11] Hanahan D, Weinberg RA (2011). Hallmarks of cancer: the next generation. Cell.

[12] Aviv A, Anderson JJ, Shay JW (2017). Mutations, cancer and the telomere length paradox. Trends Cancer.

[13] Svenson U, Nordfjall K, Stegmayr B, Manjer J, Nilsson P, Tavelin B (2008). Breast cancer survival is associated with telomere length in peripheral blood cells. Cancer Res.

[14] Gramatges MM, Telli ML, Balise R, Ford JM (2010). Longer relative telomere length in blood from women with sporadic and familial breast cancer compared with healthy controls. Cancer Epidemiol Biomark Prev.

[15] Shen J, Gammon MD, Terry MB, Wang Q, Bradshaw P, Teitelbaum SL (2009). Telomere length, oxidative damage, antioxidants and breast cancer risk. Int J Cancer.

[16] Zheng YL, Ambrosone C, Byrne C, Davis W, Nesline M, McCann SE (2010). Telomere length in blood cells and breast cancer risk: investigations in two case-control studies. Breast Cancer Res Treat.

[17] Shen J, Terry MB, Gurvich I, Liao Y, Senie RT, Santella RM (2007). Short telomere length and breast cancer risk: a study in sister sets. Cancer Res.

[18] Barwell J, Pangon L, Georgiou A, Docherty Z, Kesterton I, Ball J (2007). Is telomere length in peripheral blood lymphocytes correlated with cancer susceptibility or radiosensitivity?. Br J Cancer.

[19] Levy T, Agoulnik I, Atkinson EN, Tong XW, Gause HM, Hasenburg A (1998). Telomere length in human white blood cells remains constant with age and is shorter in breast cancer patients. Anticancer Res.

[20] Pooley KA, Sandhu MS, Tyrer J, Shah M, Driver KE, Luben RN (2010). Telomere length in prospective and retrospective cancer case-control studies. Cancer Res.

[21] Qu S, Wen W, Shu XO, Chow WH, Xiang YB, Wu J (2013). Association of leukocyte telomere length with breast cancer risk: nested case-control findings from the Shanghai Women’s Health Study. Am J Epidemiol.

[22] Kim S, Sandler DP, Carswell G, De Roo LA, Parks CG, Cawthon R (2011). Telomere length in peripheral blood and breast cancer risk in a prospective case-cohort analysis: results from the Sister Study. Cancer Causes Control.

[23] Rode L, Nordestgaard BG, Bojesen SE (2016). Long telomeres and cancer risk among 95 568 individuals from the general population. Int J Epidemiol.

[24] De Vivo I, Prescott J, Wong JY, Kraft P, Hankinson SE, Hunter DJ (2009). A prospective study of relative telomere length and postmenopausal breast cancer risk. Cancer Epidemiol Biomark Prev.

[25] Seow WJ, Cawthon RM, Purdue MP, Hu W, Gao YT, Huang WY (2014). Telomere length in white blood cell DNA and lung cancer: a pooled analysis of three prospective cohorts. Cancer Res.

[26] Yuan JM, Beckman KB, Wang R, Bull C, Adams-Haduch J, Huang JY (2018). Leukocyte telomere length in relation to risk of lung adenocarcinoma incidence: findings from the Singapore Chinese Health Study. Int J Cancer.

[27] Julin B, Shui I, Heaphy CM, Joshu CE, Meeker AK, Giovannucci E (2015). Circulating leukocyte telomere length and risk of overall and aggressive prostate cancer. Br J Cancer.

[28] Lynch SM, Major JM, Cawthon R, Weinstein SJ, Virtamo J, Lan Q (2013). A prospective analysis of telomere length and pancreatic cancer in the alpha-tocopherol beta-carotene cancer (ATBC) prevention study. Int J Cancer.

[29] Hankin JH, Stram DO, Arakawa K, Park S, Low SH, Lee HP (2001). Singapore Chinese Health Study: development, validation, and calibration of the quantitative food frequency questionnaire. Nutr Cancer.

[30] Singapore Cancer Registry Interim Report. Trends in cancer incidence in Singapore 2010–2014 [https://www.nrdo.gov.sg/docs/librariesprovider3/default-document-library/cancer-trends-2010-2014_interim-annual-report_final-(public).pdf?sfvrsn=0. Accessed July 2018.

[31] Cawthon RM (2009). Telomere length measurement by a novel monochrome multiplex quantitative PCR method. Nucleic Acids Res.

[32] Montpetit AJ, Alhareeri AA, Montpetit M, Starkweather AR, Elmore LW, Filler K (2014). Telomere length: a review of methods for measurement. Nurs Res.

[33] Harrell FE, Lee KL (1986). Verifying assumptions of the Cox proportional hazards model. Proceedings of the eleventh annual SAS user’s group international conference.

[34] WHO Expert Consultation (2004). Appropriate body-mass index for Asian populations and its implications for policy and intervention strategies. Lancet.

[35] Fordyce CA, Heaphy CM, Joste NE, Smith AY, Hunt WC, Griffith JK (2005). Association between cancer-free survival and telomere DNA content in prostate tumors. J Urol.

[36] Gadalla SM, Cawthon R, Giri N, Alter BP, Savage SA (2010). Telomere length in blood, buccal cells, and fibroblasts from patients with inherited bone marrow failure syndromes. Aging (Albany NY).

[37] Widmann TA, Herrmann M, Taha N, Konig J, Pfreundschuh M (2007). Short telomeres in aggressive non-Hodgkin’s lymphoma as a risk factor in lymphomagenesis. Exp Hematol.

[38] Daniali L, Benetos A, Susser E, Kark JD, Labat C, Kimura M (2013). Telomeres shorten at equivalent rates in somatic tissues of adults. Nat Commun.

[39] Cunningham JM, Johnson RA, Litzelman K, Skinner HG, Seo S, Engelman CD (2013). Telomere length varies by DNA extraction method: implications for epidemiologic research. Cancer Epidemiol Biomark Prev.

[40] Denham J, Marques FZ, Charchar FJ (2014). Leukocyte telomere length variation due to DNA extraction method. BMC Res Notes.

[41] Zhang C, Doherty JA, Burgess S, Hung RJ, Lindstrom S, Kraft P (2015). Genetic determinants of telomere length and risk of common cancers: a Mendelian randomization study. Hum Mol Genet.

[42] Haycock PC, Burgess S, Nounu A, Zheng J, Okoli GN, Bowden J (2017). Association between telomere length and risk of cancer and non-neoplastic diseases: a Mendelian randomization study. JAMA Oncol.

[43] Harley CB (1991). Telomere loss: mitotic clock or genetic time bomb?. Mutat Res.

[44] Varadi V, Brendle A, Brandt A, Johansson R, Enquist K, Henriksson R (2009). Polymorphisms in telomere-associated genes, breast cancer susceptibility and prognosis. Euro J Cancer.

[45] Bojesen SE, Pooley KA, Johnatty SE, Beesley J, Michailidou K, Tyrer JP (2013). Multiple independent variants at the TERT locus are associated with telomere length and risks of breast and ovarian cancer. Nat Genet.

[46] Dinami R, Buemi V, Sestito R, Zappone A, Ciani Y, Mano M (2017). Epigenetic silencing of miR-296 and miR-512 ensures hTERT dependent apoptosis protection and telomere maintenance in basal-type breast cancer cells. Oncotarget.

[47] Kyo S, Takakura M, Kanaya T, Zhuo W, Fujimoto K, Nishio Y (1999). Estrogen activates telomerase. Cancer Res.

[48] Kimura A, Ohmichi M, Kawagoe J, Kyo S, Mabuchi S, Takahashi T (2004). Induction of hTERT expression and phosphorylation by estrogen via Akt cascade in human ovarian cancer cell lines. Oncogene.

[49] Chia KS, Seow A, Lee HP, Shanmugaratnam K (2000). Cancer incidence in Singapore 1993–1997.

