# Supplementary material for: Association between prediagnostic leukocyte telomere length and breast cancer risk: the Singapore Chinese Health Study
Source: Breast Cancer Res. 2019 Apr 17;21:50. doi: 10.1186/s13058-019-1133-0 (PMC6471852; doi:10.1186/s13058-019-1133-0)
Supplement: Supplementary file 1 — Tables S1. Distributions of participants’ characteristics for women who donated blood samples for telomere length measurement and women who did not provide blood samples for telomere length measurement, the Singapore Chinese Health Study. Table S2. Associations between relative telomere length and risk of breast cancer stratified by length of follow-up, Singapore Chinese Health Study. (DOCX 21 kb) [file 13058_2019_1133_MOESM1_ESM.docx]

| **TABLE S1.** Distributions of participants’ characteristics for women who donated blood samples for telomere length measurement and women who did not provide blood samples for telomere length measurement, the Singapore Chinese Health Study | | |
| --- | --- | --- |
| **Characteristics** | **Women who donated blood samples for telomere length measurement N (%)** | **Women who did not provide blood samples for telomere length measurement N (%)** |
| **Number of participants** | 14,305 | 20,998 |
|  |  |  |
| **Age at interview, y** | 54.8 (7.5) | 57.4 (8.3) |
|  |  |  |
| **Body mass index§, kg/m^2^** |  |  |
| <18.5 | 810 (5.7) | 1297 (6.2) |
| 18.5-<23.0 | 5698 (39.8) | 7607 (36.2) |
| 23.0-<27.5 | 6488 (45.4) | 10119 (48.2) |
| 27.5+ | 1309 (9.2) | 1975 (9.4) |
|  |  |  |
| **Level of education** |  |  |
| No formal education | 4580 (32.0) | 9691 (46.2) |
| Primary school | 6034 (42.2) | 7708 (36.7) |
| Secondary school and above | 3691 (25.8) | 3599 (17.1) |
|  |  |  |
| **Alcohol consumption§ (drinks/week)** |  |  |
| None | 12831 (89.7) | 19301 (91.9) |
| < 7 | 1284 (9.0) | 1440 (6.9) |
| ≥7 | 190 (1.3) | 257 (1.2) |
|  |  |  |
| **Physical activity (weekly)** |  |  |
| No | 10421 (72.6) | 16155 (76.9) |
| Yes | 3884 (27.2) | 4843 (23.1) |
|  |  |  |
| **Smoking status§** |  |  |
| Never | 13269 (92.8) | 18929 (90.1) |
| Former/Current | 1036 (7.2) | 2069 (9.9) |
|  |  |  |
|  |  |  |
| **TABLE S1, cont'd;** | | |
| **Characteristics** | **Women who donated blood samples for telomere length measurement N (%)** | **Women who did not provide blood samples for telomere length measurement N (%)** |
| **Age when period became regular** |  |  |
| <13 | 1988 (13.9) | 2335 (11.1) |
| 13- 14 | 5152 (36.0) | 7153 (34.1) |
| 15-16 | 4578 (32.0) | 7387 (35.2) |
| 17+ | 2087 (14.6) | 3427 (16.3) |
| Never regular | 500 (3.5) | 696 (3.3) |
|  |  |  |
| **Smoking status** |  |  |
| Never | 13269 (92.8) | 18929 (90.1) |
| Former/Current | 1036 (7.2) | 2069 (9.9) |
|  |  |  |
| **Age when period became regular** |  |  |
| <13 | 1988 (13.9) | 2335 (11.1) |
| 13- 14 | 5152 (36.0) | 7153 (34.1) |
| 15-16 | 4578 (32.0) | 7387 (35.2) |
| 17+ | 2087 (14.6) | 3427 (16.3) |
| Never regular | 500 (3.5) | 696 (3.3) |
|  |  |  |
| **Age at first live birth** |  |  |
| <20.0 | 2420 (16.9) | 4431 (21.1) |
| 21.0- 25.0 | 5357 (37.5) | 8174 (38.9) |
| 26.0- 30.0 | 3962 (27.7) | 4922 (23.4) |
| 31 and over | 1526 (10.7) | 1975 (9.4) |
| Nulliparous | 1040 (7.3) | 1494 (7.1) |
|  |  |  |
| **Number of live births** |  |  |
| 0 | 1027 (7.2) | 1482 (7.1) |
| 1-2 | 4372 (30.6) | 5560 (26.5) |
| 3-4 | 5748 (40.2) | 7304 (34.8) |
| 5+ | 3158 (22.1) | 6652 (31.7) |
|  |  |  |
| **TABLE S1, cont'd;** | | |
| **Characteristics** | **Women who donated blood samples for telomere length measurement N (%)** | **Women who did not provide blood samples for telomere length measurement N (%)** |
| **Age at menopause** |  |  |
| <49 | 2425 (17.0) | 4607 (21.9) |
| 50- 54 | 3376 (23.6) | 6304 (30.0) |
| 55 and over | 8504 (59.5) | 10087 (48.0) |
|  |  |  |
| **Family history of breast cancer** |  |  |
| No | 14065 (98.3) | 20774 (98.9) |
| Yes | 240 (1.7) | 224 (1.1) |
|  |  |  |
| **Use of hormone therapy** |  |  |
| Never/Ever | 13603 (95.1) | 20399 (97.2) |
| Current | 702 (4.9) | 599 (2.9) |
|  |  |  |
| **Use of oral contraceptive** |  |  |
| Never | 9888 (69.1) | 16105 (76.7) |
| Former/Current | 4417 (30.9) | 4893 (23.3) |
|  |  |  |
| **Sleeping hours** |  |  |
| ≤6 hours | 4871 (34.1) | 7133 (34.0) |
| 7-8 hours | 8503 (59.4) | 12365 (58.9) |
| ≥ 8 hours | 931 (6.5) | 1500 (7.1) |
| Abbreviations: CI, confidence intervals; HR, hazard ratio. Values are presented as frequency (%) or mean (standard deviation). Percentages may not add up to 100% due to rounding. | | |

| **TABLE S2.** Associations between relative telomere length and risk of breast cancer stratified by length of follow-up, Singapore Chinese Health Study | | | |
| --- | --- | --- | --- |
| **Telomere length in quartile by stratification variable** | **Cases** | **Person-years** | **Adjusted HR^1^ (95% CI)** |
| **Follow-up <2 years** |  |  |  |
| Q1 (shortest) | 9 | 7072 | 1.00 (reference) |
| Q2 | 17 | 7076 | 1.90 (0.84, 4.28) |
| Q3 | 22 | 7068 | 2.44 (1.11, 5.35) |
| Q4 (longest) | 19 | 7075 | 2.13 (0.95, 4.80) |
| *P*_trend_ |  |  | 0.0641 |
|  |  |  |  |
| **Follow-up ≥2 years** |  |  |  |
| Q1 (shortest) | 75 | 35963 | 1.00 (reference) |
| Q2 | 89 | 37017 | 1.13 (0.83, 1.54) |
| Q3 | 98 | 37244 | 1.22 (0.90, 1.65) |
| Q4 (longest) | 113 | 38099 | 1.38 (1.03, 1.87) |
| *P*_trend_ |  |  | 0.0295 |
| Abbreviations: CI, confidence intervals; HR, hazard ratio. | | | |
| ^1^ Hazard ratio derived from Cox proportional hazard regression model adjusted for age at sample collection, dialect group, level of education, BMI, age when period became regular, age at first live birth, number of live births, age at menopause, use of hormone therapy, use of oral contraceptives, family history of breast cancer, smoking status, alcohol consumption, weekly vigorous work or strenuous sports, and number of hours of sleep. | | | |
